# Successional trajectory of bacterial communities in soil are shaped by plant-driven changes during secondary succession

**DOI:** 10.1038/s41598-020-66638-x

**Published:** 2020-06-17

**Authors:** Mayank Krishna, Shruti Gupta, Manuel Delgado – Baquerizo, Elly Morriën, Satish Chandra Garkoti, Rupesh Chaturvedi, Shandar Ahmad

**Affiliations:** 1 School of Environmental Sciences, Jawaharlal Nehru University, New Delhi, 110067 India; 2 School of Computational and Integrative Sciences, Jawaharlal Nehru University, New Delhi, 110067 India; 3 Departamento de Biología y Geología, Física y Química Inorgánica, Escuela Superior de Ciencias Experimentales y Tecnología, Universidad Rey Juan Carlos, C/ Tulipán s/n, 28933 Móstoles, Spain; 4 Department of Ecosystem and Landscape Dynamics, Institute of Biodiversity and Ecosystem Dynamics (IBED-ELD), University of Amsterdam, P.O. Box 94240, 1090 GE Amsterdam, The Netherlands; 5 School of Biotechnology, Jawaharlal Nehru University, New Delhi, 110067 India

**Keywords:** Ecology, Plant sciences, Ecology, Environmental sciences

## Abstract

This study investigated the potential role of a nitrogen-fixing early-coloniser *Alnus Nepalensis* D. Don (alder) in driving the changes in soil bacterial communities during secondary succession. We found that bacterial diversity was positively associated with alder growth during course of ecosystem development. Alder development elicited multiple changes in bacterial community composition and ecological networks. For example, the initial dominance of actinobacteria within bacterial community transitioned to the dominance of proteobacteria with stand development. Ecological networks approximating species associations tend to stabilize with alder growth. *Janthinobacterium lividum*, *Candidatus Xiphinematobacter* and *Rhodoplanes* were indicator species of different growth stages of alder. While the growth stages of alder has a major independent contribution to the bacterial diversity, its influence on the community composition was explained conjointly by the changes in soil properties with alder. Alder growth increased trace mineral element concentrations in the soil and explained 63% of variance in the Shannon-diversity. We also found positive association of alder with late-successional *Quercus leucotrichophora* (Oak). Together, the changes in soil bacterial community shaped by early-coloniser alder and its positive association with late-successional oak suggests a crucial role played by alder in ecosystem recovery of degraded habitats.

## Introduction

Secondary succession is the trajectory along which an ecosystem develops following disturbance events. It leads to the reestablishment of degraded sites with locally-adapted plant communities to provide stability, increase in ecosystem services and functions. Recent reports highlight the importance of considering plant- soil microbe interactions to fuel restoration efforts in a cost-effective and sustainable manner^1,2^. Certain plant species are fundamental to secondary succession, as they ameliorate microclimatic conditions and facilitate the growth of other plant species beneath their canopy^3–6^. These plants commonly known as nurse species bring emblematic shift in belowground communities and the subsequent feedbacks between nurse-soil microbiota facilitate the growth of late-successional plant species^7–9^. Moreover, the role of plant cover has recently been highlighted as a major ecological predictor of the natural changes in belowground biodiversity during ecosystem development (millions of years) globally^10,11^. Changes in soil bacterial communities driven by plant growth may influence fundamental biogeochemical processes such as pedogenesis, nutrient cycling and carbon sequestration with ecosystem development^12–18^. Some studies suggest that nurse species utilize soil microbes inhabiting the rhizosphere, which is considered a hot spot of microbial activity for faster litter decomposition and nutrient return^19–21^. This in turn exerts facilitative influence on the growth of late successional plants. Even so, the potential associations between tree growth and bacterial communities during secondary succession after disturbance remain poorly understood. Previous reports highlight that soil bacterial communities are also influenced by abiotic soil properties, geological substrate and climatic conditions^22,23^ and showed variations along soil depths^24–26^.

Here, we evaluated potential role of the growth of nitrogen-fixing tree species as a major driver of changes in bacterial community composition and diversity during ecosystem succession. It is reported that faster growing plant species which typically dominates during early-succession is associated with bacteria dominated food webs^27,28^. Bacteria dominated food-webs positively influence plant growth via faster rates of decomposition and nutrient cycling. Therefore, investigation of potential association between soil bacterial community and early-colonizer plants become crucial for ecosystem development^29,30^. Focusing on these ideas, we investigated the potential facilitative role of an early- colonizer plant species *Alnus nepalensis* (Himalayan alder), on the changes in soil bacterial communities and on potential association with late-successional plant species during secondary succession. Himalayan alder belongs to the widely distributed genus Alnus which comprises of c. 35 species widely distributed in northern hemisphere including boreal, temperate and montane tropical climatic regimes^31^. Alder species are found mostly in degraded habitats with nutrient impoverished conditions in the soil^32^. These plant species have a symbiotic association with *Frankia* which allows alder roots to fix atmospheric nitrogen^33^. It is reported that alder during primary succession in glacier forefield shapes the soil bacterial community structure and subsequent feedbacks between alder-soil bacteria may help in establishment and colonization by late-successional plant species^29,30,34^. Alder also produces cluster roots which secrete carboxylates and mobilize available phosphorus and other mineral elements in the soil^35^. Moreover, recent reports on alder^36^ establish its role in accelerating rock-weathering and thereby supplying multiple nutrients that may limit carbon fixation in a forest ecosystem. In Himalaya, alder is used for traditional agroforestry purposes and is also endorsed for the recovery of degraded and abandoned sites^37,38^. However, a critical gap exists regarding the alder-soil bacterial interactions and the role of alder in affecting soil bacterial communities during secondary succession. We hypothesize that (1) Alder enriches the nutrient-depleted sites with nitrogen and other essential soil nutrients, and improves the soil properties. (2) Alder growth significantly shapes the soil bacterial community and its interaction with soil bacteria may further play an important role in the recovery of degraded sites (3) Suitable niches created by alder would lead to the establishment and growth of late-successional plant species. To test these hypothesis, our objectives are to (1) investigate the variation in the soil nutrients and mineral elements along a chronosequence of alder stand development (2) study the changes in bacterial diversity, community structure, species interactions within bacterial community affected by alder growth (3) assess the possibility of coexistence of alder with late-successional plant species.

## Materials and Methods

### Study area and sampling design

The study sites were located between 30°31′36.7′′N and 30°32′34.5′′N, and 79°6′42.0′’E and 79°07′23.9′′E in the Rudraprayag district of Garhwal division of Uttarakhand state of India. The study sites were in proximity of Kedarnath wildlife sanctuary as a part of reserve forest. Mean monthly temperature varies between 7**°**and 36 °C and area annually receives 300 mm/year precipitation on average with monsoon season (mid-June to mid-Sept) accounting for three-fourth of annual rainfall. The topography of the area has historically been influenced by landslides/slips that are common during the monsoon season. Three landslides affected sites, where *Alnus nepalensis* was at different stages of growth were selected for detailed study. All the sites were located at similar elevation at 1400 ± 60 m, with similar slope, aspects (North-east slopes) and with a road distance of approximately Two km. Three alder stands were designated as Juvenile, Young and Mature based on the growth stages of alder individuals in the stands. These stands were selected for understanding plant- soil bacterial community interactions and examining the influence of alder growth on the soil bacterial communities. In the juvenile stand, individuals of alder were in seedling stage and in young and mature stand, trees were with average diameter at breast height (DBH) 54 ± 15 cm and 115 ± 10 cm, respectively representing stand age as 0–2, 25–30 and 45–60 years. Further, to understand the influence of alder on the growth of late successional plant species, additional sites where alder coexisted with *Quercus leucotrichophora* (Oak) were selected.

Soil cores at 0–10 cm,10–20 cm, 20–30 cm depth in triplicate from each site and at a distance interval of 5 meters were collected from the rhizosphere in a S-shaped pattern and were mixed to form a composite sample^39–41^. Pooling of the soil samples was done to account for within-plot spatial heterogeneity^42^. It is important to note that our main question was not to understand the dynamics of bacterial communities shaped in soil conditioned by alder vs. bare soil, but rather, to evaluate the temporal changes in bacterial communities with alder development. As alder usually grows in pure patches and in our study, we made sure that the soils were collected from such sites only, for understanding the alder driven changes. The selected sites, were located in close proximity, exposed to similar climatic conditions, land use history, and were affected by landslides in the past. In our opinion, this precaution should allow us to answer our research question.

Soils were divided into two subsets and were transported to the laboratory immediately. One subset of soil samples from each site was carried in sterile plastic bags and was stored in −80° for metagenomic analysis and other subset was used for soil physicochemical characterization.

### Soil physicochemical characterization and major element geochemistry

The subset of soil samples collected for soil physicochemical parameters were sieved to <2 mm, weighted and dried at 105 °C to a constant weight. These soil samples were used for the determination of soil pH, carbon (C), nitrogen (N), phosphorus (P) concentrations along with the analysis of the major element geochemistry in soil samples.

Soil organic carbon was estimated through modified Walkley and Black oxidation method^43^. Soil nitrogen was estimated through micro-kjeldhal digestion procedure^44^. Soil pH was measured on 1:2.5 diluted soil solution.

Determination of oxides of major elements i.e. Iron (Fe), Manganese (Mn), Magnesium (Mg), Calcium (Ca), Sodium (Na), Potassium (K), Phosphorus (P), Aluminium (Al) for soils were accomplished through WD- X-Ray Fluorescence Spectrometry (XRF, PANalytical. Axios) following standard methods^45,46^.

### DNA extraction and purification

Metagenomic DNA was isolated from the soil samples (0.25 g) by using Nucleospin soil kit. The quality and quantity of the extracted DNA (1 µl) was measured in nanodrop for determining A260/A280 ratio. DNA sample was further processed for first amplicon generation followed by NGS library preparation using Nextera XT index kit (Illumina Inc.) preparation kit. The mean of the library fragment size distribution varied from 573 bp to 599 bp. The libraries were sequenced on MiSeq using 2 × 300 bp chemistry. Primers for the amplification of the 16S rDNA gene (16SrRNAF–GCCTACGGGNGGCWGCAG and 16S rRNA R- ACTACHVGGGTATCTAATCC) specific for bacteria were designed at Eurofins genomic lab facility (Karnataka, India). Amplification for the 16S rDNA gene targeting bacteria was carried out. 4 µl of PCR product was resolved on 1.2% agarose gel at 120 V for approximately 60 min or till the samples reached 3/4^th^ of the gel.

### Bioinformatics analysis

Processing of the 16S rRNA derived sequence inventories was performed using QIIME^47^. Briefly, using Cutadapt^48^ we trimmed the sequencing primers from the forward and reverse reads. Read pairs were removed if both the forward and reverse primers were not detected (10% mis-matches were allowed for primer search). Paired-ends were merged, demultiplexed and the sequences were stitched into single end reads in QIIME (v1.9)^49^. The operational taxonomic units (OTUs) based on sequence similarities within the reads were picked up. All the sequences from the sample was clustered into operational taxonomic units (OTUs) based on their sequence similarity. OTUs are clusters of sequences, frequently intended to represent some degree of taxonomic relatedness done using UCLUST at 97% sequence similarity after removal of chimeras using USEARCH software (and each resulting cluster typically represents a species. Since each OTU may be made up of many sequences, we picked a representative sequence for the downstream analysis of that out following removal of singletons. The representative sequence was used for taxonomic identification of the OTU by setting assignment method to the RDP (Ribosomal database project) classification system and a bootstrap threshold of 0.8^50^. Based on OTU tables community dissimilarity matrices using the weighted UniFrac method were calculated in QIIME^51^.

### Heat map and ecological network of soil bacterial community

Comparative analysis between soil samples was performed at different taxonomic level setting a threshold of 0.5% abundance. The 50 most abundant operational taxonomic units (OTUs) among the soil samples were chosen for depth wise analysis using hierarchical clustering based on Euclidean distance (complete linkage) in MeV version 4.8^52^. By depth-wise analysis, we wanted to observe similarities or possible trends in OTUs abundance for same depths with the successive growth of alder. The heat map provided a better visual outcomes by displaying the relative abundances of bacterial communities across soil samples. Further, microbial co-occurrence networks using OTUs were constructed to get an insight to the potential bacterial association within the soil^53^. For this a cut-off of 0.5% abundance^24,54,55^ in at least one depth for each stand were fixed. Although microbial co-occurrence networks have traditionally been derived using Spearman correlation of relative abundance between two microbial genera^24,56,57^ the soil samples were collected from few available sites, making correlations less meaningful and uncertain with small sample size^58^. Euclidean distance as one of the simplest metric was used to derive a similarity log score matrix and were used for constructing microbial networks. Resulting adjacency matrix was plotted using the Fruchterman-reingold layout of the interactive platform Gephi version 0.9.2, where nodes are coloured by phylum and size of the node is determined by the median percentage abundance of the OTUs in all depth in an alder stand. The topological properties of the resulting network were described using a set of measures (nodes, edges, clustering coefficient, average path length, diameter, modularity) using network 2.0 Python module. Further, to access the robustness of the network, we generated 1000 random networks for each age with same number of nodes. We calculated the mean of number of connections i.e. edges^54^. Mean of the number of edges from these graphs were treated as expected scores in the chi-square test.

Relative influence of each taxon within the networks was described using betweenness centrality values of each OTU. BC value of each OTU was ranked and OTUs within the top ten percentile in each network were used to identify the keystone species for each network^55,59,60^.

### Statistical analysis

An analysis of variance (ANOVA) was performed to assess the significance of the effect of alder growth and soil depth on the soil properties using PAST version 3.19. We also performed a one-way analysis of similarity (ANOSIM) test with Bray-Curtis distance^61^ to analyse the extent of variation in species composition with the growth of alder. A similarity percentage (SIMPER) procedure^61^ was used to elucidate the contribution of each species to temporal changes in species composition. Spearman’s correlation analysis was performed to evaluate the relationship between soil properties and alder growth and microbial communities. Correlation analysis was performed in R version 3.4.2 (R Core Team 2017). Redundancy analysis (RDA) was performed with CANOCO Version 4.5 to understand the effect of soil nutrients on the relative abundance of different bacterial phyla. Linear regression analysis quantified relationship between Shannon diversity and organic carbon and Iron as well as abundance of Proteobacteria and organic carbon. Further, The variance partitioning analysis was used to quantify the contribution of four factors i.e. growth stages of alder, soil properties (C, N, P) which showed significant variations with alder development, elements which changed significantly and elements which showed non-significant changes with alder development on bacterial diversity and community composition. Variance partitioning analysis was performed using the varpart function in the vegan package in R version 3.4.2^23^. In particular, this analysis provided insights on whether changes seen in bacterial diversity and community composition is influenced by alder growth or due to changes in soil properties.

## Results

### Changes in soil properties and climax plant species during secondary succession of alder

We found important changes in soil properties (at three depths i.e. 0–10 cm,10–20 cm, 20–30 cm) with stand development during secondary succession (Table S1). Except for potassium (K), significant variation (p ≤ 0.05) was observed in soil nutrients with the growth of alder (Table S2). Except for Ca, K, Mg, Fe, soil nutrients and properties such as organic carbon, nitrogen, phosphorus, sodium varied significantly (p ≤ 0.05) along soil depths (Table S2). Soil pH was relatively stable varying (4–5.13) across all sites. Alder was found to grow both as pure stand as well as with other plant species in Himalaya. At the sites where it coexists with other species, a linear positive association between the growth of alder with coexisting late-successional plant species *Quercus leucotrichophora* was found (Fig. S1).

### Changes in bacterial diversity, community composition and network assembly during alder stand development

**Figure 1:**
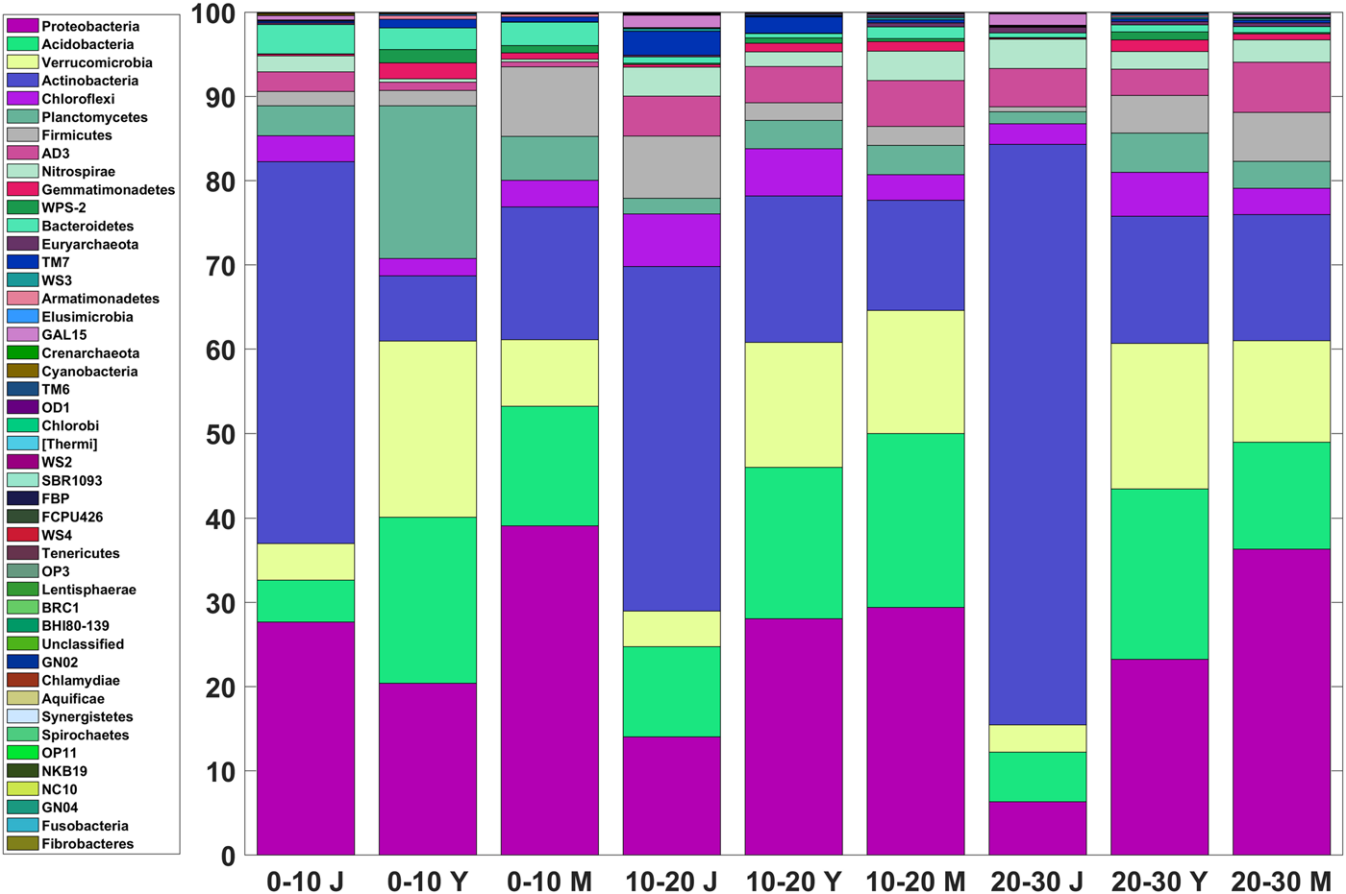
Comparative analysis between soil samples representing successive depths of different stages of alder growth at phylum level.

A total of 31,908 operational taxonomic units (OTUs) at 97% similarity were found in the soil samples with number of OTUs varying from 2,604 to 4,475 (see Fig. S2 for rarefaction curve & Table S3 for the most abundant taxonomy identified at different taxonomic levels). We found drastic changes in the community composition of bacterial communities during secondary succession of alder stand development. Analysis of soil bacterial community composition revealed sequences belonging to 47 phyla, 144 classes, 282 orders, 450 families, 813 genera, 985 species in soil samples across all the stands (Fig. S3). However, the dominant phyla common to all sites included Actinobacteria, Proteobacteria, Acidobacteria, Verrucomicrobia, Planctomycetes, Chloroflexi, Firmicutes accounting for almost 80% of the bacterial sequences (Fig. 1). Additionally, AD3, Nitrospirae, Bacteroidetes, TM7, Gemmatimonadetes, WPS2 were also present in the soil samples with varying proportions across the sampling sites.

Shannon diversity increased to more than two folds from 4.21 (minimum) at the juvenile stand to 9.34 (maximum) at the mature stand. Maximum bacterial diversity were found in the top 0–10 cm across all successional stages (Table S3). The comparison of bacterial diversity in the soil samples suggest a vertical decline in Shannon diversity with depth across all sites (Table S3).

Unlike Actinobacteria, phylotypes within Proteobacteria, Verrucomicrobia, Acidobacteria increased during secondary succession (Fig. 1). The changes in bacterial composition with the age of alder stands was further revealed by the analysis of similarity (Anosim R = 0.67., P = .0067). A similarity percentage (SIMPER) procedure revealed 34% dissimilarity at the phylum level (Table S4). In terms of contribution, the phylum Actinobacteria, Proteobacteria, Verrucomicrobia, Acidobacteria, Planctomycetes and Firmicutes cumulatively contributed around 85% of the total microbial dissimilarity (Table S4).

**Figure 2:**
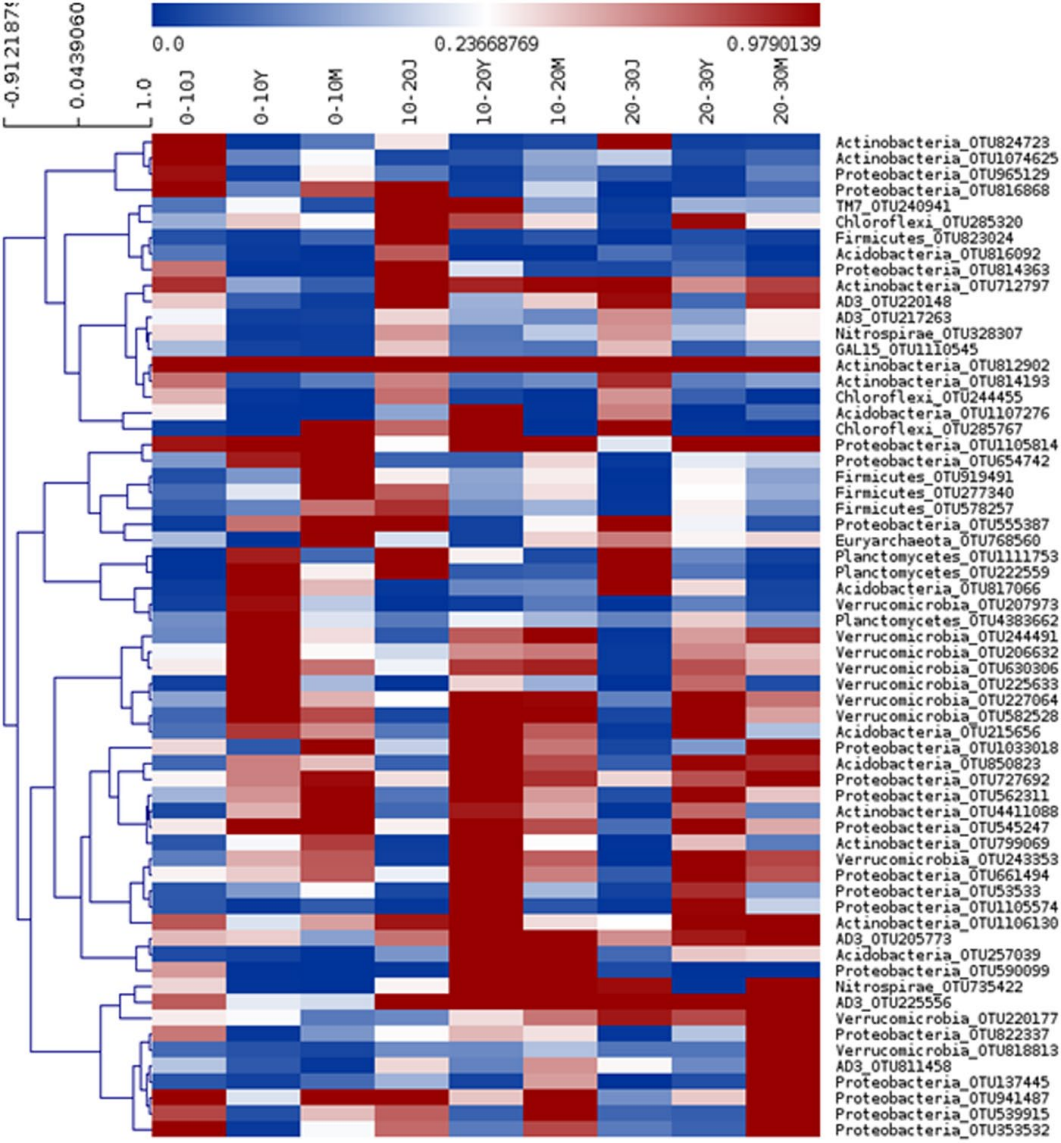
A Heat map diagram accompanying hierarchical clustering based on the relative abundance of top 50 OTUs showed for successive depth at different stages of Aader growth. The colour scale represents the normalized value of relative abundance of OTUs.

Heat maps showed that OTUs affiliated to the same taxonomic groups had similar responses during succession (Fig. 2). OTUs associated with Actinobacteria showed a decreasing trend of relative abundance both with depth, and along stand age (Fig. 2). OTUs affiliated with Proteobacteria and Verrucomicrobia were prominent in the soils associated with young and mature alder stands. OTUs associated with Firmicutes showed higher relative abundance in the top layer of mature soil (Fig. 2). Overall there was a gradual shift in dominance from Actinobacteria towards Proteobacteria and Verrucomicrobia.

### Influence of alder on the interactions within co-occurring soil bacterial taxa

**Figure 3:**
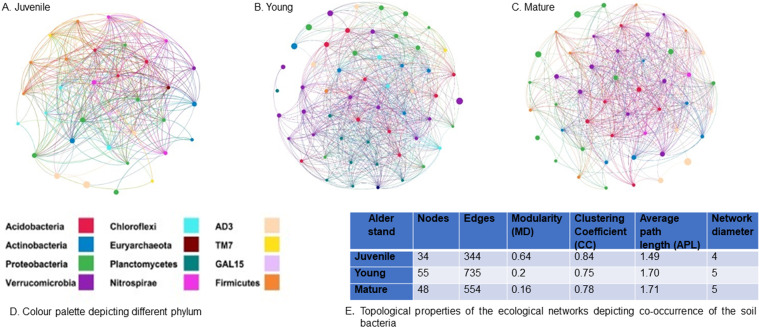
Bacterial co-occurrence network analysis: Networks of co-occurring bacteria at different stages of alder growth (**A**) Juvenile (**B**) Young (**C**) Mature. Nodes of the network are coloured by phylum with size of the node corresponds to relative abundance of bacterial phyla. (**D**) Colour palettes representing different phyla (**E**) Topological properties of the bacterial networks.

Microbial co-occurrence networks provide an insight to the potential associations within the bacterial community wherein individual taxa are associated with each other either directly or indirectly through intermediate species^53,54,56^. Our results suggest that alder growth influences the potential bacterial associations during succession (Fig. 3). At the juvenile stage, edges which is a proxy for the number of connections and the average path length were 344 and 1.49 respectively, which was less than 735, 1.70 at the young and 554,1.71 at the mature stage, respectively suggesting strong association among soil bacteria facilitated by alder (Fig. 3E). Further, number of edges in the randomly generated networks varied significantly at young and mature stages of alder (Table S5). Another factor that represents a subnetwork within a network, wherein species are more interconnected relative to the species outside the module^62,63^ decreased consistently with stand development (Fig. 3E). Modularity was maximum at the juvenile stage, with 0.64 suggests modular structure of the network which decreased to 0.2, 0.16 at the young and the mature stages of alder stand respectively. Betweenness centrality score which is a proxy for elucidating the keystone taxa found to differ at different stages of alder growth. OTUs describing keystone taxa were affiliated to the phyla Proteobacteria, Actinobacteria, Verrucomicrobia, Acidobacteria and AD-3 (TableS 6). Keystone species that were identified in the present study were *Janthinobacterium lividum*, *Candidatus Xiphinematobacter*, *Rhodoplanes* at the juvenile, young and mature alder stand respectively (Table S6).

### Key ecological drivers of bacterial diversity and community structure in the soil samples during succession

**Figure 4:**
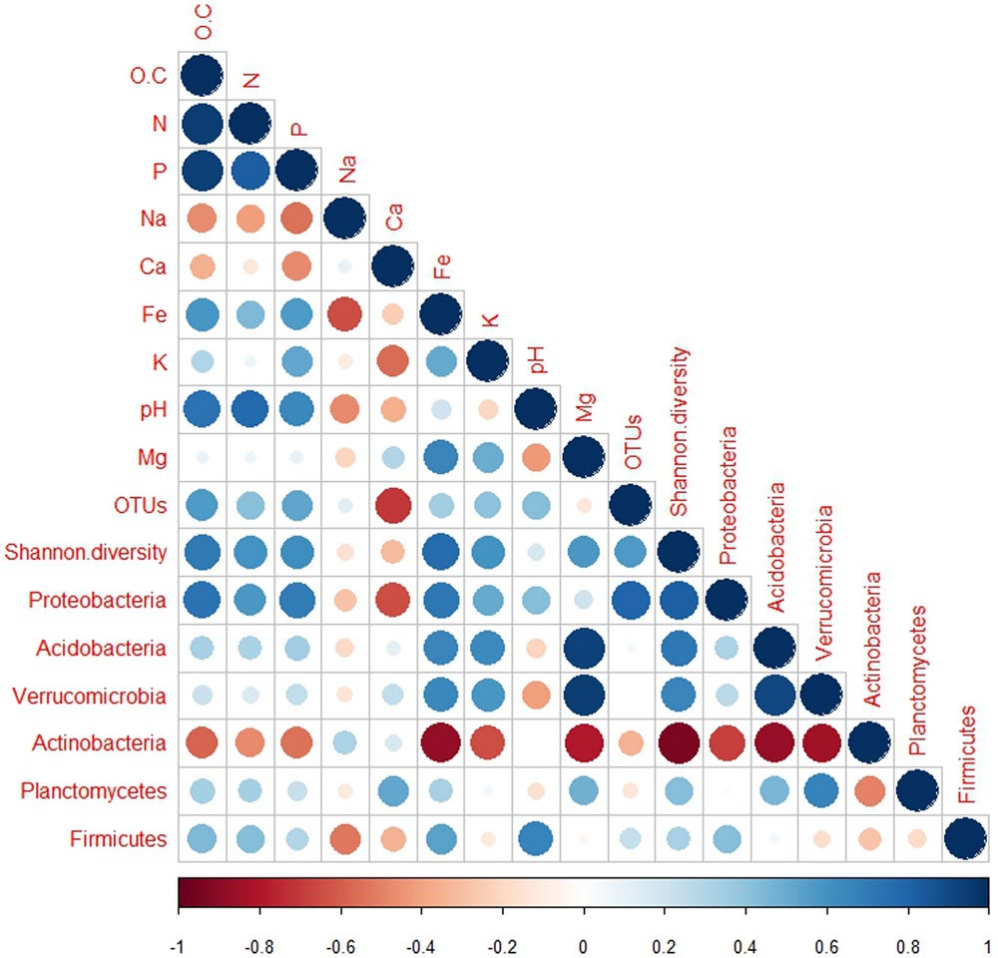
Correlation matrix between soil nutrient changed with alder growth and distribution of bacterial phyla in the soils.

**Figure 5:**
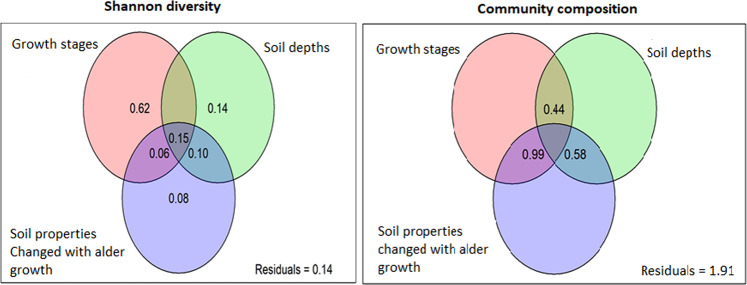
Relative contribution of alder growth, soil depths and (**A**) soil properties changed with alder growth (**B**) Mineral elements changed significantly with alder growth (**C**) Mineral elements showed non-significant changes with alder as predictors of bacterial diversity. Panels represent results from variation partitioning models  to identity the percentage variance of bacterial diversity with different predictor variables. Shared effects of these variable groups are indicated by the overlap of circles.

We found that multiple environmental variables influenced the relative abundance of bacterial phyla significantly during forest succession (p ≤ 0.05). Among the elements that significantly changed with the stand development, concentration of organic carbon, nitrogen, phosphorus and iron were significantly correlated with the Shannon-diversity and Proteobacteria (Fig. 4). These elements were also negatively correlated with the Actinobacteria along with the magnesium. Magnesium was found to be positively correlated with the Acidobacteria and Verrucomicrobia. Further, Shannon diversity showed negative correlation with Actinobacteria (Fig. 4). Shannon diversity was positively albeit weakly correlated with soil organic carbon concentration (r^2^ = 0.49, p = 0.03) (Fig. S4(A)) and iron (r^2^ = 0.59, p = 0.015) (Fig. S4(B)). True to its copiotrophic character, the distribution of proteobacteria was found significantly influenced by the availability of organic carbon (r^2^ = 0.53; p = 0.02) (Fig. S4(C)). Redundancy analysis (RDA) revealed that edaphic variables explained 80.3% variance, with axis-1 and axis-2 explaining 67.5% and 12.8% of total variance in soil bacterial abundance (Fig. 5), respectively. Concentrations of Iron, organic carbon, nitrogen and magnesium had positive effect on the bacterial phyla Proteobacteria, Firmicutes and Bacteriodetes. Bacteria affiliated to the phylum Actinobacteria Euryachaeota, Nitrospirae, AD3, WS3, formed a close association which may be ascribed to be influenced by similar edaphic factors (Fig. S5).

The Variation partitioning indicated that while the stages of alder development explained a unique portion of the variance in the Shannon-diversity, its influence on the community composition at OTU was not exclusive (Fig. 5(A,B). Additionally, alder along with soil elements such as Na, Ca, Fe, which changed with stand development together explained about 63% of variance in the Shannon diversity (Fig. S6).

## Discussion

Our study provides evidence that changes in bacterial diversity and community composition are associated with ecosystem succession. These changes in bacteria are directly or indirectly (changes in soil properties) associated with alder growth after landslide disturbance. Alder growth had a positive effect on bacterial diversity and elicited multiple changes in the relative abundance of bacterial phyla. Some of these changes might be the consequence of alder growth changing the environment. For example, due to nitrogen fixation, higher litter inputs with alder growth and the release of carboxylates by cluster roots, alder may have enriched the nutrient-depleted substratum with carbon and other limiting nutrients, more importantly, nitrogen and phosphorus^29,35,64^. The organic matter and other nutrient inputs provided by alder have manifested in increase in bacterial diversity and community composition. These changes in bacterial community may in turn lead to the supply of trapped mineral elements. However with a small sample size which is a caveat associated with the present study, the influence of other potential factors as a driver of soil bacterial diversity confounding with alder growth may not be fully negated. There is a need to assess the possible role played by other factors in addition to alder growth in driving the successional dynamics of the soil bacteria. The increase in mineral elements in the soils with alder stand development is in consonance with the recent report^36^ on another species but of same genus alder wherein it accelerated nutrient inputs from the rock weathering. This may also be explained by the previous reports which ascribed the role of cluster roots^29^ in promoting the nutrient mobilization in the soils by actinorhizal plants. Moreover, creation of microsites beneath the canopy of alder possibly have allowed sufficient moisture retention in addition to nitrogen fixation and improvement in soil properties^38^. All these effects of alder on abiotic soil properties might explain the larger richness of bacteria observed with time. The amelioration of stressed habitat conditions imposed after landslides by alder also positively influenced the growth of *Quercus leucotrichophora* (oak) which has been able to attain greater growth in dense shade^65^. The changes in abiotic soil properties by alder and its positive manifestation on the growth of late-successional plant species further supplement the finding of previous research of its role during primary succession^29,30,34,64^. Niche separation between an early-coloniser alder and late-successional oak species, due to the variation in ecophysiological traits may allow coexistence of both these species. Similar positive effects of alder on the successful establishment and growth of late-successional *Picea sitchensis* during primary succession have been reported in glacier forefield^29,30^. Moreover, results from present study is in consonance with the findings of the recent reports wherein plant cover has been seen as a major ecological predictor of natural changes in belowground biodiversity during ecosystem development (millions of years) globally^11^.

Our results further support a transposition from an Actinobacteria dominated bacterial community at the juvenile stand to Proteobacteria dominated at later stages of alder stand development. In the soil bacterial community, a major contribution by the phyla Actinobacteria, Proteobacteria, Verrucomicrobia, Acidobacteria, Planctomycetes and Firmicutes is consistent with the previously reported bacterial community composition from forest soils globally, more specifically from alder-conditioned soils^30,39,41,66^. Previous reports^41^ based on experimental and meta-analysis have categorized certain soil bacteria phyla into *copiotrophs and oligotrophs* analogous to the r‐ and K‐selected categories often used to describe the ecological attributes of plants and animals. Such a copio-oligotrophic concept may also help us to understand our results.

Actinobacteria are often dominant in drylands where they thrive under low nutrient and dry conditions^67^. They are also an important decomposers of woody debris often accumulated after landslides^68,69^. Actinobacteria degrades dead woody-mass and supply carbon to the associated mycorrhizae and thereby may play an important role as mycorrhizae helper bacteria^70^. Proteobacteria on the other hand, are mostly represented by fast-growing copiotrophs that are adapted to high carbon and nutrient availability^39,41^. Our results also showed a positive response of soil Proteobacteria to an increase in soil organic carbon similar to previous reports^71^. The increase in soil bacterial diversity and shifts in dominance towards phylotypes associated with Proteobacteria for an e.g. genus Bukholoderia with successive alder growth may help in rock weathering and releasing mineral elements such as Iron^17,22,72^. The shift in in the dominance of oligotrophs to copiotrophs induced by alder growth corresponds to a pattern that is mostly observed aboveground among plant communities wherein k-selected species are gradually replaced by r-selected species with nitrogen fertilization^73^. The transposition of oligotrophs to copiotrophs with improvement in soil nutrients is in line with previous reports^24,39,40,66^. The increase in bacterial diversity and dominance of the taxa affiliated to Proteobacteria at later stages of succession may play an important role in nitrogen cycling and may complement alder in the nitrogen fixation^16^, coupling iron-carbon biogeochemistry^74^, carbon sequestration, nutrient turnover, and other biogeochemical processes^15^.

In addition to bacterial diversity and changes in bacterial composition, microbial co-occurrence networks approximating the species associations are getting stabilised with the alder stand development. Although our networks deviated significantly from random networks, we acknowledge that the statistical significance is difficult to establish due to the small number of samples. However, there are enough leads from our study which needs further investigation. Changes driven by alder growth on the bacterial association was revealed by the topological properties of the ecological networks at different stages. Alder growth manifested changes in network properties such as decreasing clustering coefficient, increasing path length and also a slight increase in the average diameter of the network. As the modular networks are supposedly more efficient^63^ the decrease in modularity in our results may be attributed to the increasing dominance of copiotrophs with low C-use efficiency with the growth of alder. The keystone genera, which have a significant influence on the community structure differ at all stages. In the present case, it was affiliated to the phylum Proteobacteria, Actinobacteria, Verrucomicrobia, AD3, and Acidobacteria which is consistent with previously reported results^55^. *Janthinobacterium lividium* emerged as one of the keystone species in present study is in consonance with the previous reports on the bacterial community from alder-conditioned soils during primary succession^29^. The difference in keystone taxa with the growth of alder suggests a possible realignment in species interaction within the bacterial community.

## Conclusions

Taken together, our study provides initial leads about the role played by alder in shaping soil bacterial diversity, community composition and potential associations within the bacterial community during secondary succession. Proteobacteria dominating at later stages of succession may further supplement the process of nitrogen fixation by alder. The ecological implications of alder growth and soil bacteria is supported by potential direct (unique explained variation) and indirect (e.g., shared variation with soil properties) effects of alder growth on bacterial diversity and community composition. Addition of soil nutrients, changes in soil bacteria driven by alder and its positive association with late-successional oak supports the crucial role played by alder in ecosystem recovery of degraded sites bypassing mid-successional stages of succession. Given the continent-wide distribution of alder, further work focusing the mechanisms that tie both- above and below ground changes driven by alder may infer its potential role for future restoration efforts.

## Supplementary information

Supplementary Information.
