# Publisher Correction: Successional trajectory of bacterial communities in soil are shaped by plant-driven changes during secondary succession

**DOI:** 10.1038/s41598-020-68560-8

**Published:** 2020-07-07

**Authors:** Mayank Krishna, Shruti Gupta, Manuel Delgado – Baquerizo, Elly Morriën, Satish Chandra Garkoti, Rupesh Chaturvedi, Shandar Ahmad

**Affiliations:** 1 School of Environmental Sciences, Jawaharlal Nehru University, New Delhi, 110067 India; 2 School of Computational and Integrative Sciences, Jawaharlal Nehru University, New Delhi, 110067 India; 3 Departamento de Biología y Geología, Física y Química Inorgánica, Escuela Superior de Ciencias Experimentales y Tecnología, Universidad Rey Juan Carlos, C/ Tulipán s/n, 28933 Móstoles, Spain; 4 Department of Ecosystem and Landscape Dynamics, Institute of Biodiversity and Ecosystem Dynamics (IBED-ELD), University of Amsterdam, P.O. Box 94240, 1090 GE Amsterdam, The Netherlands; 5 School of Biotechnology, Jawaharlal Nehru University, New Delhi, 110067 India

Correction to: *Scientific Reports* https://doi.org/10.1038/s41598-020-66638-x, published online 17 June 2020

This Article contains a typographical error the Author Contributions section.

“M.K., S.C.G., R.C. designed research, M.K. performed research, M.K., S.C.G., S.A., M.D., E.M. analysed data, S.C.G., R.C. contributed new reagents and analytical tools, M.K. wrote the paper with inputs from all authors.”

should read:

“M.K, S.C.G, R.C designed research, M.K. performed research, M.K, S.G, M.D, E.M, S.A analysed and interpreted the data, R.C, S.C.G contributed new reagents, M.K wrote the paper with inputs from all authors.”

