# Supplementary material for: Successional trajectory of bacterial communities in soil are shaped by plant-driven changes during secondary succession
Source: Sci Rep. 2020 Jun 17;10:9864. doi: 10.1038/s41598-020-66638-x (PMC7299987; doi:10.1038/s41598-020-66638-x)

Successional trajectory of bacterial communities in soil are shaped by plant-driven changes during secondary succession

Authors

Mayank Krishna[1], Shruti Gupta[2], Manuel Delgado – Baquerizo[3], Elly Morriën[4], Satish Chandra Garkoti[1$], Rupesh Chaturvedi[5], Shandar Ahmad[2]

Affiliations

1. School of Environmental Sciences, Jawaharlal Nehru University, New Delhi-110067, India

2. School of Computational and Integrative Sciences, Jawaharlal Nehru University, New Delhi-110067, India

3. Departamento de Biología y Geología, Física y Química Inorgánica, Escuela Superior de Ciencias Experimentales y Tecnología, Universidad Rey Juan Carlos, C/ Tulipán s/n, 28933 Móstoles, Spain

4. Department of Ecosystem and Landscape Dynamics, Institute of Biodiversity and Ecosystem Dynamics (IBED-ELD), University of Amsterdam, P.O. Box 94240, 1090 GE Amsterdam, The Netherlands.

5. School of Biotechnology, Jawaharlal Nehru University, New Delhi-110067, India

Table S1: Variation in soil nutrients and properties with Alder growth along three successive depths. Value in each depth represents the mean ± standard error (n=9).

Alder growth stage	Depth (cm)	pH	N (%)	O.C (%)	P (%)	Na (%)	Ca (%)	K (%)	Mg (%)	Fe (%)
**Juvenile**	0-10	4.29 ±.004	0.146 ± .003	3.39 ± .29	0.16 ± .008	1.21 ± .11	0.71 ± .014	3.76 ± 0.17	2.26 ± 0.50	5.003 ± 1.81
	10-20	4.38 ± .001	.126 ± .003	2.47 ± .29	0.19 ± .006	0.82 ± .02	1.13 ± .06	3.91 ± .15	4.02 ± .23	5.97 ± .23
	20-30	4.35±.001	.116 ± 0.005	1.92 ± .25	0.25 ± .03	0.66 ± 0.09	0.75 ± .06	3.75 ± .16	2.94 ± .17	6.20 ± .29
**Young**	0-10	4.14±.006	0.30 ± .005	5.14 ± .14	0.11 ± .003	0.89 ± 0.05	0.85 ± .07	3.64 ± .07	2.81 ± .06	5.36 ± .34
	10-20	4.04±.003	.183 ± .003	2.97 ± .10	0.14 ± .03	0.93 ± 0.04	0.93 ± .03	3.93 ± .26	3.97 ± .41	5.71 ± .15
	20-30	4.17±.003	.153 ± .006	2.87 ± .08	0.23 ± 0.008	0.94 ± 0.07	0.58 ± .08	4.55 ± .32	3.49 ± .24	5.78 ± .15
**Mature**	0-10	5.13±.004	0.58 ± 0.003	8.64 ± .28	0.14 ± .003	0.83 ± 0.83	1.01 ± .11	3.58 ± .14	2.49 ± .31	4.78 ± .79
	10-20	4.32±.001	0.283 ±0.003	4.56 ± .12	0.13 ± .03	0.94 ± 0.05	0.93 ± .004	3.97 ± .36	4.15 ± .53	6.09 ± .17
	20-30	4.37±.001	.153 ± .003	3.88 ± .09	0.20 ± .02	0.59 ± 0.03	0.57 ± .08	4.20 ± .37	3.38 ± .04	6.51 ± .11

Table S2: Summary of two -way Anova for the different soil properties incorporating the main effect of growth stages and depths. Highlighted numerical values represents significant difference.

**Soil nutrient**		df	F	**p-value**
O.C (%)	Growth stages	2	25.14	**6.15E-06**
	Depth	2	13.65	**0.000247**
N (%)	Growth stages	2	17.58	**2.30E-21**
	Depth	2	16.62	**3.81E-21**
P (%)	Growth stages	2	19.37	**3.26E-05**
	Depth	2	4.554	**0.02509**
Na	Growth stages	2	14.41	**0.000184**
	Depth	2	4.944	**0.01944**
Ca	Growth stages	2	15.82	**0.000108**
	Depth	2	0.698	0.5106
K	Growth stages	2	3.174	0.06598
	Depth	2	0.6473	0.5352
Mg	Growth stages	2	16.66	**8.02E-05**
	Depth	2	0.9727	0.3971
Fe	Growth stages	2	3.818	**0.04149**
	Depth	2	0.1127	0.8941

Table S3 : Comparative analysis between soil samples and most abundant taxonomy identified in the samples at different taxonomic levels

**Sample statistics**	**0-10(J)**	**10-20(J)**	**20-30(J)**
**OTUs**	4,475	2,728	2,604
**Shannon-diversity**	7.54	6.79	4.21
**Phylum**	Actinobacteria(45.4)	Actinobacteria(40.87)	Actinobacteria(68.88)
**Class**	Actinobacteria(42.25)	Actinobacteria(37.93)	Actinobacteria(67.8)
**Order**	Actinomycetales(42.24)	Actinomycetales(37.93)	Actinomycetales (67.38)
**Family**	Micrococcaceae(34.5)	Micrococcaceae(35.31)	Micrococcaceae(66.03)
**Genus**	Arthrobacter (32.42)	Arthrobacter (31.77)	Arthrobacter (62.67)
**Sample statistics**	**0-10(Y)**	**10-20(Y)**	**20-30(Y)**
**OTUs**	2,922	3,068	3,854
**Shannon-Diversity**	8.92	8.38	8.27
**Phylum**	Verrucomicrobia (20.88)	Protobacteria (28.07)	Protobacteria (20.88)
**Class**	Spartobacteria (18.89)	Alphaproteobacteria (16.21)	Spartobactertia (16.13)
**Order**	Chthoniobacteria (18.89)	Chthoniobacteria (14.16)	Chthoniobacteria (16.13)
**Family**	Chthoniobacteraceae (18.89)	Chthoniobacteraceae (14.16)	Chthoniobacteraceae (16.13)
**Genus**	DA101 (13.65)	DA101 (11.97)	DA101 (14.07)
**Sample statistics**	**0-10(M)**	**10-20(M)**	**20-30(M)**
**OTUs**	4,343	4,050	3,864
**Shannon-Diversity**	9.34	9.09	8.3
**Phylum**	Proteobacteria (39.11)	Proteobacteria (29.4)	Proteobacteria (36.31)
**Class**	Alphaproteobacteria (24.78)	Spartobacteria (13.61)	Beta-proteobacteria (23.35)
**Order**	Bukhoderiales (22.1)	Rhizobiales (14.96)	Chthoniobacteriales (13.61)
**Family**	Oxalobacteraceae (20.96)	Hyphomicrobiaceae (7.68)	Chthnobacteriaceae (13.61)

**Genus**	Unclassified (14.9)	Rhodoplanes (6.67)	DA 101 (10.73)

Table S4: SIMPER analysis deciphering microbial dissimilarity among the sampling sites revealed total dissimilarity of 33.58%.

Taxon	Average dissimilarity	Contribution (%)	Cumulative %
Actinobacteria	10.97	32.68	32.68
Proteobacteria	6.13	18.26	50.93
Verrucomicrobia	3.802	11.32	62.26
Acidobacteria	3.58	10.66	72.92
Planctomycetes	2.226	6.63	79.55
Firmicutes	1.614	4.807	84.36
AD3	1.14	3.396	87.75
Chloroflexi	0.854	2.543	90.3
Nitrospirae	0.7329	2.183	92.48
Bacteroidetes	0.6318	1.881	94.36
TM7	0.5056	1.506	95.87
Gemmatimonadetes	0.3584	1.067	96.93
GAL15	0.3025	0.901	97.83
WPS-2	0.2948	0.8778	98.71
Euryarchaeota	0.1392	0.4145	99.13
Armatimonadetes	0.07612	0.2267	99.35
WS3	0.07223	0.2151	99.57
Cyanobacteria	0.0275	0.08191	99.65
SBR1093	0.0275	0.08191	99.73
Elusimicrobia	0.02611	0.07777	99.81
Crenarchaeota	0.02084	0.06205	99.87
TM6	0.007501	0.02234	99.89
OD1	0.006667	0.01986	99.91
GN04	0.004723	0.01407	99.93
Chlamydiae	0.004167	0.01241	99.94
NC10	0.003334	0.009928	99.95
FBP	0.002778	0.008274	99.96
Chlorobi	0.0025	0.007446	99.97
WS2	0.001945	0.005792	99.97
FCPU426	0.001945	0.005792	99.98
Tenericutes	0.001945	0.005792	99.98
Thermi	0.001945	0.005792	99.99
Unclassified	0.001111	0.003309	99.99
OP3	0.001111	0.003309	100
Fusobacteria	0.001111	0.003309	100

Table S5: Robustness analysis of microbial co-occurrence networks: Random similarity matrices were created for each age with values in the range of original networks. Number of edges for the randomly generated (1000) networks for each age were taken as expected score in the chi-square test. Significant differences is highlighted in red.

Stand age	Nodes	Edges (in original networks)	Edges (in random networks)	Chi-square test
**Juvenile**	**38**	**344**	**333.64 ± 13.3**	**0.57**
**Young**	**58**	**735**	**1117 ± 19.29**	**0.00**
**Mature**	**51**	**554**	**727.17 ± 17.55**	**0.00**

Table S6: Variations in keystone taxa calculated on the basis of betweenness centrality score of the OTUs along the alder growth.

	OTU ID	B.C	Phylum	Species
Juvenile	822337	0.069	Proteobacteria	Janthinobacterium lividum
	814363	0.051	Proteobacteria	
	814193	0.049	Actinobacteria	
	1074625	0.06	Actinobacteria	
Young	244491	0.074776	Verrucomicrobia	Candidatus Xiphinematobacter
	4411088	0.058822	Actinobacteria	Unclassified
	545247	0.037154	Proteobacteria	Unclassified
	630306	0.037037	Verrucomicrobia	Unclassified
	557983	0.035565	Verrucomicrobia	Unclassified
	215656	0.028728	Acidobacteria	Unclassified
Mature	1106130	0.134907	Actinobacteria	
	822337	0.076098	Proteobacteria	Janthinobacterium;lividum
	661494	0.059182	Proteobacteria	Rhodoplanes
	205773	0.043236	AD3	
	712797	0.034192	Actinobacteria	

Fig. S1. Linear correlation between the growth of alder (*Alnus nepalensis*) and Oak (*Quercus leucotrichophora*) tree species. Each points represents sampling sites where alder coexists with oak species.

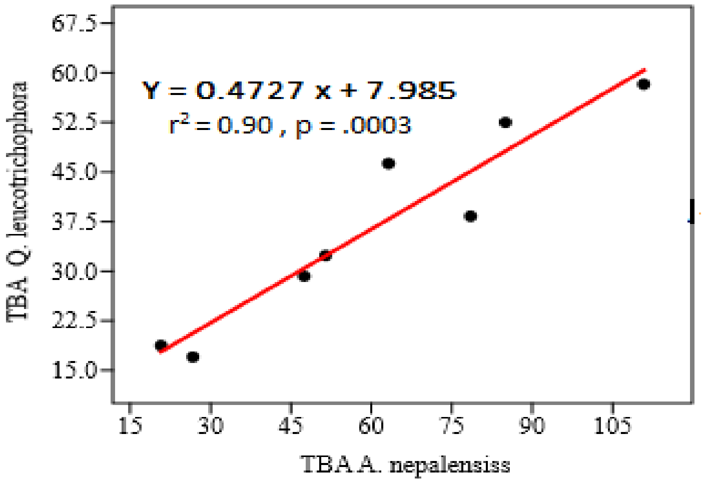

Fig. S2: The rarefraction curves for the soil samples

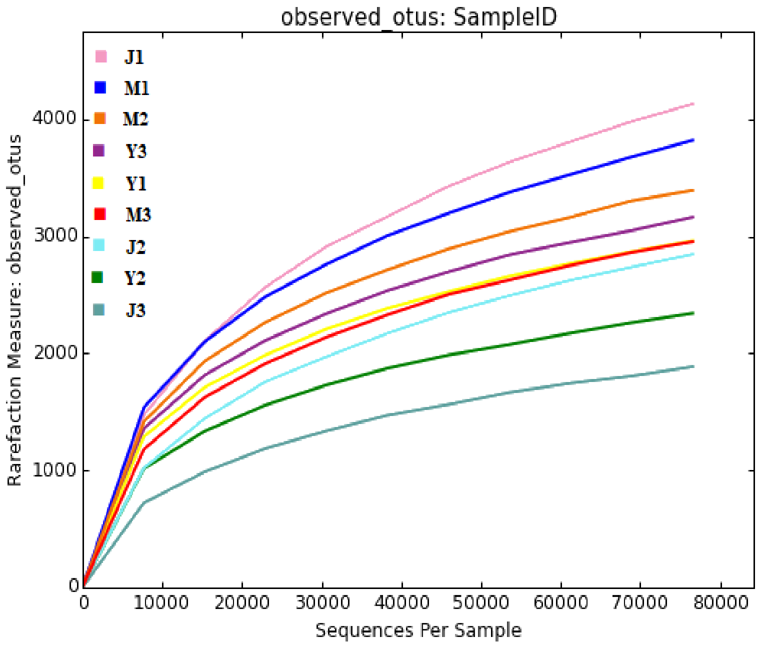

Fig. S3: Pie charts depicting the Absolute abundance at phylum level within each bacterial community

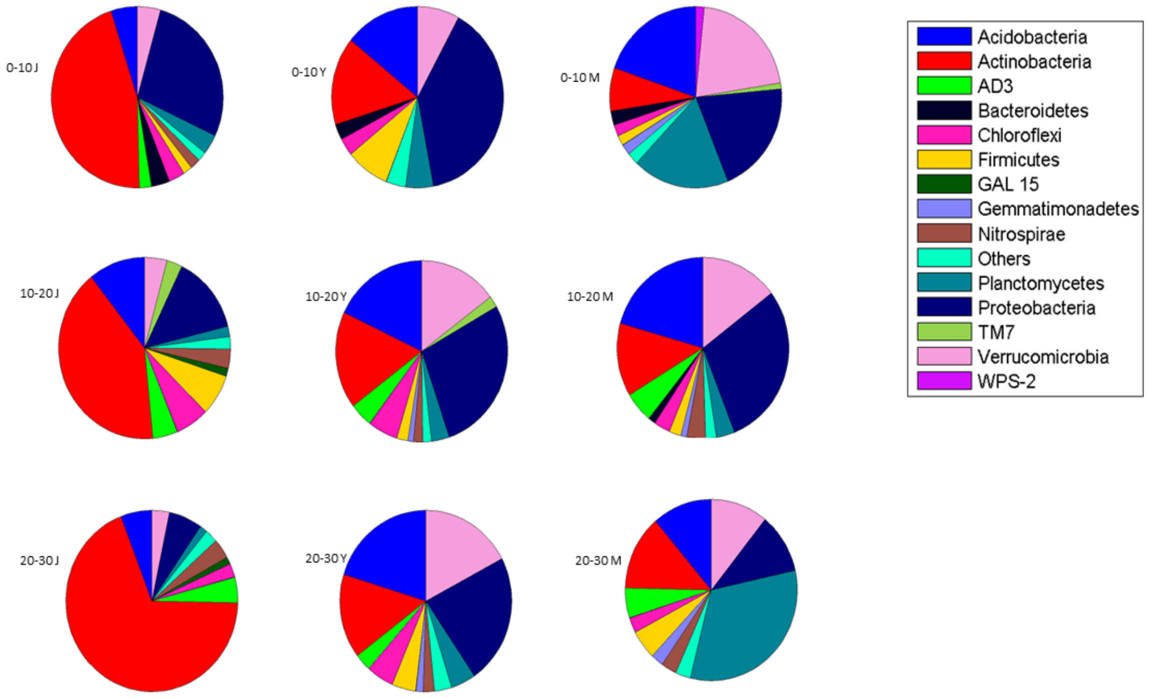

Fig. S4: Linear correlation between (A) Shannon diversity and percentage organic carbon in soil samples (B) Shannon diversity and Percentage iron is soil samples (C) Absolute abundance of Proteobacteria phyla and percentage organic carbon in soil samples. Linear regression is shown for each figures with their levels of significance. Each point (n= 9) represents sampled soils

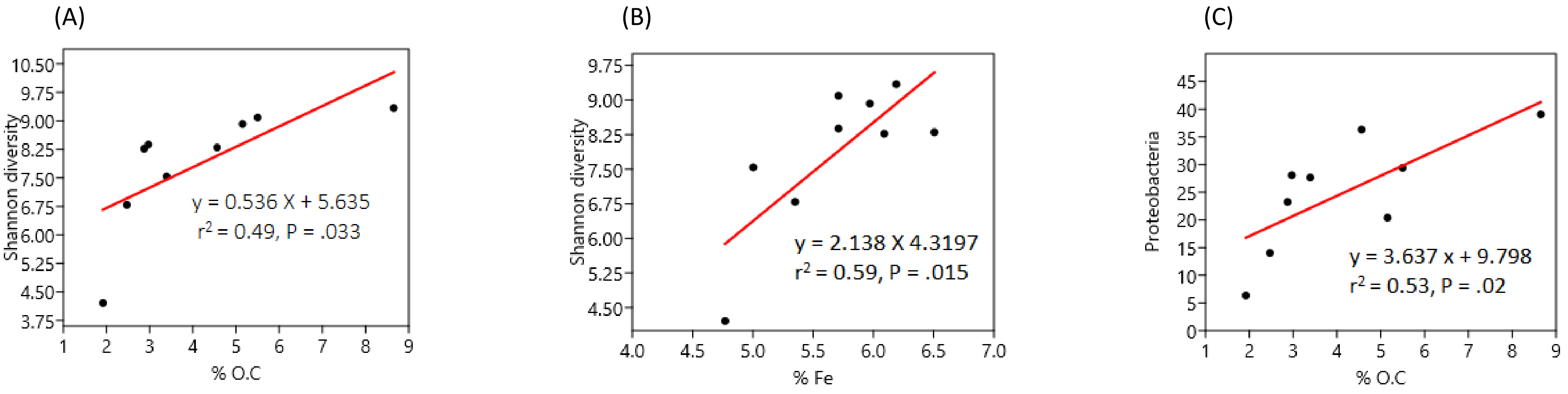

Fig. S5: Redundancy analysis of soil properties and bacterial community in the soils sampled from the different aged alder stands

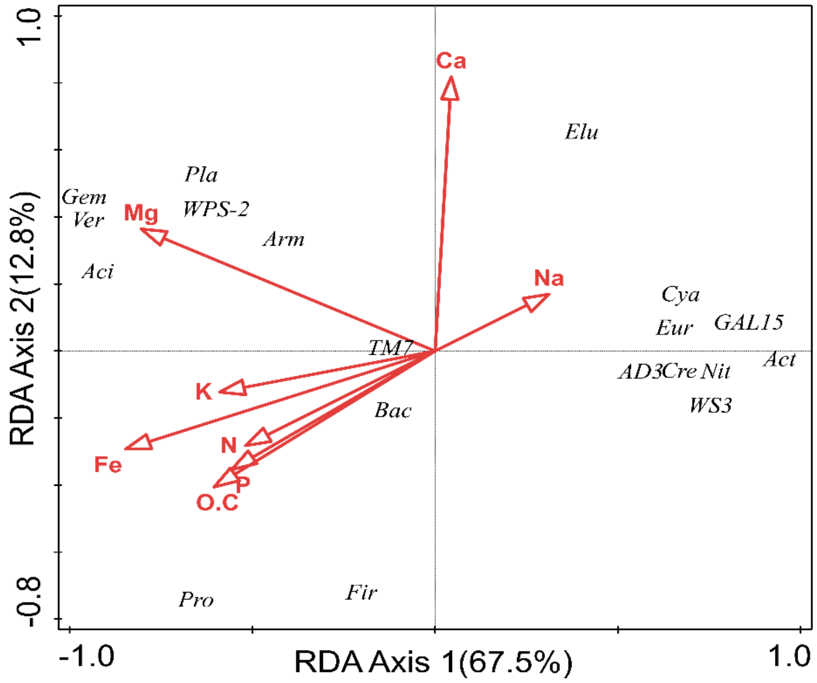

Fig. S6: Relative contribution of alder growth, soil depths and mineral elements that changed significantly and those elements which showed non-significant changes with stand development as predictors of bacterial diversity and community composition at the OTU level. Panels represent results from variation partitioning modeling aiming to identity the percentage variance of bacterial bacterial diversity and community composition explained by growth stages, soil depths and mineral elements changing showing significant and non-significant changes with stand development. Shared effects of these variable groups are indicated by the overlap of circles

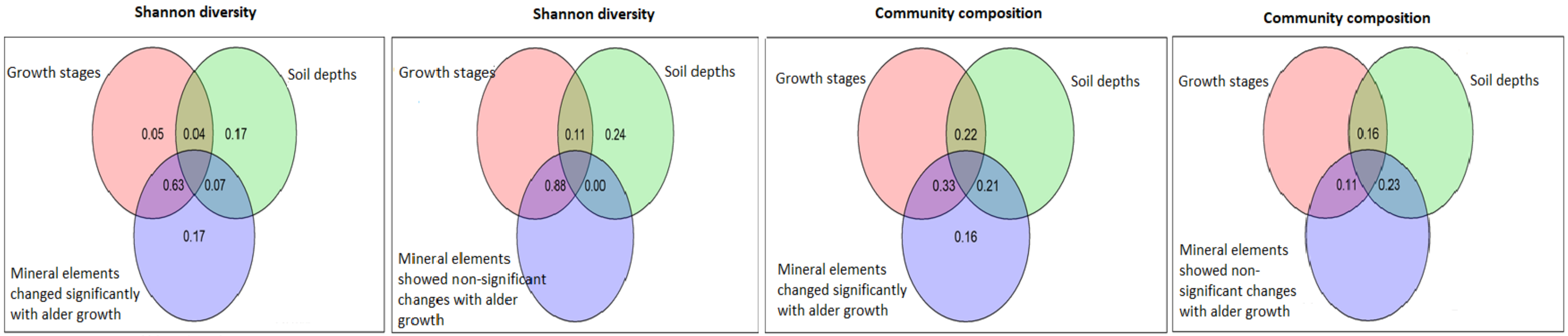

Supplement: Supplementary file 1 — Supplementary Information. [file 41598_2020_66638_MOESM1_ESM.pdf]
